# Impact of Information Technology on Information Gaps in Canadian Ambulatory Care Encounters

**DOI:** 10.2196/medinform.4066

**Published:** 2015-01-08

**Authors:** Lauren Korosec, Krista Balenko, Simon Hagens

**Affiliations:** ^1^Ipsos-Reid CorporationToronto, ONCanada; ^2^Canada Health InfowayToronto, ONCanada

**Keywords:** digital health, information gaps, ambulatory, outpatient

## Abstract

**Background:**

Specialist physicians require clinical information for patient visits in ambulatory encounters, some of which they may access via digital health solutions.

**Objective:**

This study explored the completeness of information for patient care and the consequences of gaps for ambulatory specialist services provided in ambulatory settings in Canada.

**Methods:**

A sample of specialist physicians practising in outpatient clinics was recruited from a health care provider research panel. The study (n=1800 patient encounters) looked at the completeness of patient information experienced by physicians who work in environments with rich health information exchange (Connected) and a comparison cohort with less information available electronically (Unconnected).

**Results:**

Unconnected physicians were significantly more likely to be missing information they needed for patient encounters (13% of encounters for Unconnected physicians vs 7% for Connected physicians). Unconnected physicians were also more likely to report that missing information had consequences (23% vs 13% of encounters). Lab results were the most common type of patient information missing for both Unconnected and Connected specialists (25% for Unconnected physicians vs 11% Connected physicians).

**Conclusions:**

The results from this study indicate that Canadian physicians commonly experience information gaps in ambulatory encounters, and that many of these gaps are of consequence to themselves, their patients, and the healthcare system. Wasting physician and patient time, as well as being forced to proceed with incomplete information, were the most common consequences of information gaps reported.

## Introduction

Ambulatory care includes a wide range of health care services for patients who are not admitted overnight to a hospital. These services are performed at outpatient clinics, urgent care centers, ambulatory or same-day surgery centers, diagnostic and imaging centers, primary care centers, community health centers, occupational health centers, mental health clinics, and group practices. Canadian Institute for Health Information reported 34 million hospital ambulatory care service visits across all provinces (excluding Quebec) in 2011-2012 [1]. The vast majority of these patients would also receive care in other settings, such as from primary care providers, making continuity and coordination of care a priority.

Evidence suggests that information availability in ambulatory care settings is an important factor for productivity and quality of care [2-5]. Some research has looked at the impact of information technology and health information exchange on addressing ambulatory care information gaps in specific settings [6-9] with generally positive findings, but there is a need for further research in the Canadian context on the impacts of information technology in these diverse and complex settings.

Canadian hospitals, health authorities, and governments are making significant investments in both local digital health solutions and broader health information exchanges, with increased adoption resulting from these investments. In 2006, there were approximately 7600 users of electronic health records that share information across settings. By 2014, this had increased significantly to over 62,000 users across Canada [10]. Primary Care Physician use of electronic medical records in their practices increased from 24% in 2007 to 64% in 2013 [11].

This study explored the completeness of information for patient care and the consequences of gaps for ambulatory specialist services provided in ambulatory settings in Canada. The completeness of information is based on the extent of information available to physicians electronically from within their practice setting through their own electronic medical record and beyond through access to jurisdictional electronic health record services.

## Methods

A sample of 18 specialist physicians practicing in outpatient clinics was recruited from a health care provider research panel. Physicians who do not see patients in an ambulatory setting, those who never require lab or diagnostic imaging results and those who were not from the nine target specialty groups were removed from the sample. Those that remained were segmented into three groups based upon their reported use of information technology in their main ambulatory setting:

1. “Connected” physicians (63% of qualifying physicians with n=9 recruited for this study) had access to and used comprehensive digital health solutions within their practice settings, such as electronic medical records or hospital information systems. For example, 8 of 9 physicians have entered encounter notes electronically and 9 of 9 could electronically view lab and diagnostic imaging reports that they had ordered. They also had access to patient information from outside their practice setting through health information exchanges (8 of 9 for lab results, 9 of 9 for diagnostic imaging reports, 9 of 9 for a full medication history, and 8 of 9 for referral notes).
2. “Unconnected” physicians (15% of qualifying physicians with n=9 recruited for this study) had similar access to internal digital health solutions (7 of 9 could access lab and diagnostic imaging reports for tests that they ordered), but only 1 of 9 entered clinical notes electronically. They had less external connectivity, with 1 of 9 able to access lab data from other providers or settings and 1 out of 9 with electronic access to referral notes.
3. “Partially connected” physicians (22% of qualifying physicians with none included in this study) had some use of digital health use within their practice setting.

This distribution is generally consistent with the variations in digital health use in Canadian ambulatory clinics. Access to internal hospital systems for laboratory and diagnostic imaging test results is now common across the country, with varying levels of progress in implementing health information exchanges in different regions [12]. For example, as shown in Table 1, while the 9 Connected physicians are from across the country, there is a heavier concentration in the western Canada. There was also a mix of specialist types across the two study groups.

**Table 1:** Specialty/region matrix for Connected and Unconnected physicians.

Region		IT Enabled				Non-IT Enabled			
	
		West	Ontario	Quebec/East	Total	West	Ontario	Quebec/East	Total
**Specialty**									
	Cardiology						1	2	3
	General Internal Medicine	1	1		2			1	1
	Endocrinology						2		2
	Nephrology								
	Oncology		1	1	2				
	Ophthalmology	1			1	1		1	2
	Orthopedics						1		1
	Surgery	1	2	1	4				
	Urology								
Total		3	4	2	9	1	4	4	9

The 18 physicians that participated in this study collected data about information needs, gaps, and impacts at each of 100 distinct patient visits randomly selected using Canada Health Infoway’s Patient Data Collection form for a total of n=1800 individual patient encounters. For each encounter, this form was used to capture the need for patient information such as lab results, diagnostic images and reports, medications and referral/clinical notes, the completeness of that information, and the consequences of any information gaps. Differences between the Connected and Unconnected groups were evaluated using a *t* test, *P*=.05, as calculated using Quantum 5.8.

Physicians collected data between July 17 and August 12, 2013. They were offered an incentive of around CAN $500 for 100 completed forms. Ethics approval was obtained from the Sudbury and District Health Unit Research Ethics Review Committee.

## Results

Physicians reported that one or more types of patient information were required for almost all of the 1800 ambulatory clinic visits tracked in this study. As shown in Table 2, clinical/referral notes were most often needed (95% of encounters), followed by lab results and medications at 71% and 67% respectively. Immunization information was least often needed of the 8 types of information included in the Patient Data Collection Form (required in 3% of encounters).

**Table 2:** Proportion of all encounters for which a specific type of information was required.

Information Needed		
	Base	Proportion %
Clinical/Referral Notes	1715	95%
Lab Results	1280	71%
Medications	1211	67%
Diagnostic Imaging	1060	59%
Allergies	750	42%
Discharge/ED Reports	531	30%
Specialist Referral/Appointment Status	300	17%
Immunizations	62	3%
All Encounters Total	1800	100%

**Figure 1:**
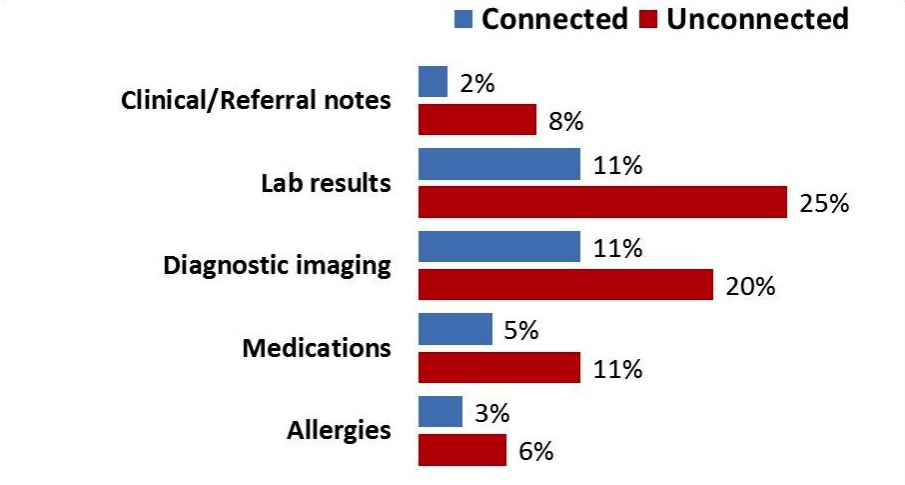
Missing information by required patient information.

Connected physicians were more likely than Unconnected physicians to report having the patient information they needed during clinical encounters. This was true across all of the five types of information most often required (see Figure 1). The largest differential between the two groups was missing lab results (25% Unconnected vs 11% Connected) or diagnostic imaging test results (20% Unconnected vs 11% Connected).

In addition to analyzing results by Unconnected versus Connected, New Patient encounters versus Regular Patient encounters were also explored. At an overall level, information gaps of any type were more common for patients new to the physician (15% of such encounters), than patients the physicians have previously seen (7% of encounters).

**Figure 2:**
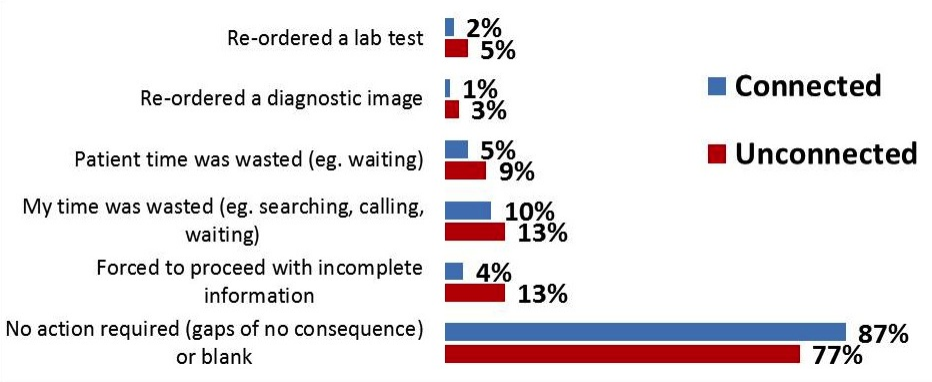
Impacts of missing information.

Information gaps for Connected physicians were more likely to result in “no action required”, or there was no consequence to the missing information, compared to information gaps for Unconnected physicians (see Figure 2). Across the 900 encounters in the Connected group, 87% of the time, physicians either indicated “no action required” or identified no impacts related to information gaps. That compares to 77% of encounters for the Unconnected group.

There were statistically significant differences in the impacts of information gaps between the two groups for five of the six types of potential impacts investigated. It is also important to note that consequences of information gaps existed for both groups, Connected and Unconnected; however, Unconnected physicians were more likely to have to take action because of the missing information than Connected physicians.

Physician time was more likely to be wasted because of information gaps with Unconnected physicians (13%) compared to Connected physicians (10%). Likewise, as a result of missing information, patient time was more likely to be wasted for Unconnected physician encounters (9%) compared to Connected physician encounters (5%). While re-ordering of tests was less common for both groups, the Unconnected group was significantly more likely to report re-ordering a lab test or diagnostic imaging because of information gaps (5% and 3% of encounters respectively), compared to 2% and 1% of encounters with Connected physicians, respectively. With over 34 million ambulatory care encounters in Canada annually, even differences of 2-3% in test volumes are meaningful and substantive in terms of daily patient volume, costs, and impact on the patient. The largest differential between Connected versus Unconnected physicians is where physicians indicated they were forced to proceed with incomplete information, which impacted 4% of encounters for the Connected group and 13% for the Unconnected group.

### Limitations

While the sample size in terms of physicians (n=18) across a diverse group of specialties is a limitation of this study, the total number of encounters studied (n=1800) provides a robust basis for analysis. Due to the sample size, we were not able to match specialist types or geographic regions across the two groups. Potential diversity in information needs across specialty types is mitigated by focusing on gaps only for information types that physicians report being required in each encounter. It should also be noted that the existence of information gaps and the related impacts were subject to interpretation by the participating physicians.

## Discussion

The number of patients being seen in ambulatory clinics is rising, both in Canada [13] and in other countries. These patients tend to be receiving care in multiple settings from a variety of providers, making care coordination important. Investments in local digital health solutions and broader health information exchange aim to help address this challenge.

The results from this study indicate that Canadian physicians commonly experience information gaps in ambulatory encounters, and that many of these gaps are of consequence to themselves, their patients, and the healthcare system. However, Connected physicians (those who indicate they have more robust internal and external electronic access to patient information) are much less likely to experience information gaps compared to Unconnected physicians. Information gaps experienced are also more likely to have a material impact for Unconnected physicians and their patients. The findings thus support the drive to increase availability and adoption of digital health solutions and health information exchange in order to provide authorized clinicians with more complete information to support patient encounters.

This study also generates some important questions for further research. While having access to electronic information from both inside and outside the organization reduce the incidence of information gaps and related impacts, there are still 13% of encounters in the Connected group where action was required or the gaps had other consequences. A better understanding of the source of these gaps will be important for continuing to improve health care quality, including coordination and continuity of care. Likewise, for both groups it would be helpful to understand the magnitudes of time wasted, implications of being forced to proceed with incomplete information, and consequences of having to re-order laboratory or diagnostic imaging tests.
